# Improved fluorescent *Listeria* spp. biosensors for analysis of antimicrobials by flow cytometry

**DOI:** 10.1002/mbo3.1304

**Published:** 2022-06-30

**Authors:** Sebastian J. Reich, Jonas Stohr, Oliver Goldbeck, Bastian Fendrich, Peter Crauwels, Christian U. Riedel

**Affiliations:** ^1^ Institute of Microbiology and Biotechnology University of Ulm Ulm Germany

**Keywords:** antimicrobials, biosensors, fluorescence, *Listeria*, single‐cell analysis

## Abstract

The global increase in antibiotic resistance of pathogenic microorganisms requires the identification and characterization of novel antimicrobials. Bacterial biosensors expressing fluorescent proteins such as pHluorin variants are suitable for high‐throughput screenings. Here, we present *Listeria* spp. pH‐sensitive biosensors with improved fluorescence for single‐cell analysis of antimicrobials by flow cytometry.

## Graphical abstract

For screening of novel antimicrobial compounds, we developed improved *Listeria* spp. biosensor strains. By using pH‐dependent fluorescence, a quick readout of the integrity status of the cell is possible. We further demonstrate the assessment of cell integrity of the sensor bacteria on the single‐cell level by flow cytometry.

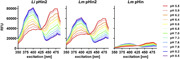

1

The increasing global challenges with (multi)drug‐resistant bacteria highlight the demand for novel antimicrobial compounds to treat life‐threatening infections (World Health Organization, 2014). Despite this growing need for novel anti‐infective agents, the number of new antibiotics on the market is steadily decreasing (Theuretzbacher et al., 2020; Towse et al., 2017). A major bottleneck in the development of new antimicrobial drugs is the lack of rapid, cost‐effective, and reliable screening tools for lead compound identification (Miethke et al., 2021). Recently, our group has developed live a biosensor of the food‐borne pathogen *Listeria monocytogenes* for the detection of antimicrobial compounds that kill target bacteria by pore formation and disruption of membrane integrity (Crauwels et al., 2018). The biosensor is based on monitoring intracellular pH by expression of the green fluorescent protein‐derivative pHluorin, which is characterized by two distinct excitation peaks that change in relative fluorescence intensities in response to pH (Miesenböck et al., 1998). These biosensors were successfully used to determine the susceptibility of bacteria to the lantibiotic nisin, measure antimicrobial activity in supernatants for natural and recombinant producers of antimicrobial peptides, and screen a library of bacteria isolated from raw milk for producers of antimicrobials (Desiderato et al., 2021; Goldbeck et al., 2021; Weixler et al., 2022).

Similar to the previously published biosensor strain *L. monocytogenes* EGDe/pNZ‐P_help_‐pHluorin (*Lm* pHin), a new vector was constructed, in which the pHluorin gene was replaced with a gene for pHluorin2, a pHluorin derivative with enhanced fluorescence (Mahon, 2011). The backbone of pNZ44 (McGrath et al., 2001) was linearized by restriction with *Bgl*II and *Pst*I (FastDigest enzymes, Thermo Fisher Scientific) to remove the p44 promoter. The strong, constitutive P_help_ promoter was amplified from pPL2*lux*P_help_ (Riedel et al., 2007) using primers P_help__fw (TTTTTATATTACAGCTCCAATCATTATGCTTTGGCAGTTTATTC) and P_help__rv (CTTTACTCATGGGTTTCACTCTCCTTCTAC) using Q5 polymerase (New England Biolabs) and a standard PCR protocol with 61.9°C annealing temperature and 15 s elongation time. The gene encoding pHluorin2 was obtained as a synthetic DNA fragment codon‐optimized for *L. monocytogenes* by a commercial service provider (Eurofins Genomics) and amplified using primers pHin2LM_fw (GTAGAAGGAGAGTGAAACCCATGAGTAAAGGTGAAGAATTATTTAC) and pHin2LM_rv (AGTGGTACCGCATGCCTGCACTATTTATATAATTCATCCATACCATGTG) (Q5 polymerase, 58.6°C annealing, 45 s elongation). Vector backbone and PCR products were assembled in a single isothermal reaction as described by Gibson et al. (2009). Relevant parts of the resulting plasmid pNZ‐pHin2^*Lm*^ (Figure 1a) were verified by Sanger sequencing (Microsynth Seqlab). Correct plasmids were used to transform *L. monocytogenes* EGDe and *Listeria innocua* LMG 2785 by electroporation using a previously described protocol (Monk et al., 2008) and positive clones were selected on brain heart infusion (BHI) agar containing 10 µg/ml chloramphenicol. Following successful transformation, biosensor strains carrying plasmids pNZ‐P_help_‐pHluorin or pNZ‐pHin2^*Lm*^ were initially checked for fluorescence by imaging in an iBright^TM^ FL1000 Imaging System (Thermo Fisher Scientific) with fluorescence detection mode at 488 nm (Figure 1b).

**Figure 1:**
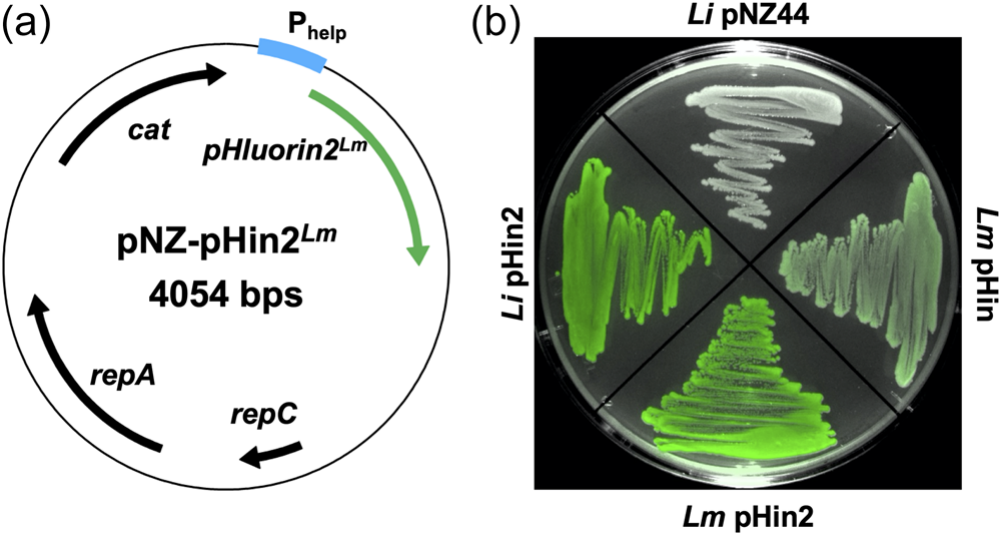
(a) Plasmid map of pNZ‐pHin2^*Lm*^ with *pHluorin2* codon‐optimized for *Listeria monocytogenes* under control of the strong, constitutive P_help_‐promoter, *repC* and *repA* encode replication proteins, and *cat* for a chloramphenicol acetyltransferase used for selection. (b) Fluorescence of *Listeria innocua* LMG 2785/pNZ44 (*Li* pNZ44), LMG 2785/pNZ‐pHin2^*Lm*^ (*Li* pHin2), *L. monocytogenes* EGDe/pNZ‐pHin2^*Lm*^ (*Lm* pHin2), or EGDe/pNZ‐P_help_‐pHluorin (*Lm* pHin) imaged in an iBright FL1000 in an overlay (photograph and fluorescence detection mode at 488 nm).

Both new strains containing pNZ‐pHin2^*Lm*^ (*Li* pHin2, *Lm* pHin2) showed brighter fluorescence on agar plates than the previously published strain *Lm* pHin, whereas the empty vector control strain *L. innocua* LMG 2785/pNZ44 (*Li* pNZ44) showed no fluorescence above background.

To further analyze the fluorescence properties of the biosensors, bacteria were grown in BHI overnight (i.e., approx. 16 h), washed once in phosphate‐buffered saline (PBS), and adjusted to an OD_600_ of 3 in filter‐sterilized (pore size 0.2 μm) *Listeria* minimal buffer (LMB) (Crauwels et al., 2018) adjusted to different pH (5.5–8.5). Aliquots of 100 μl were distributed into single wells of a black microtiter plate and mixed with 100 μl of LMB containing the cationic detergent cetyltrimethylammonium bromide (final concentration 0.002% w/v) for membrane disruption. After incubation for 30 min at room temperature, fluorescence excitation spectra (350–490 nm) were recorded at an emission wavelength of 520 nm using a Tecan Infinite® M200 multimode plate reader (Tecan).

Similar to the previously published *Lm* pHin (Crauwels et al., 2018), *L. innocua* LMG 2785/pNZ‐pHin2^*Lm*^ (*Li* pHin2) and *L. monocytogenes* EGDe/pNZ‐pHin2^*Lm*^ (*Lm* pHin2) displayed the typical excitation spectrum of pHluorin proteins with excitation peaks at 400 and 475–480 nm (Figure 2). All three strains also showed the characteristic ratiometric, pH‐dependent shift in fluorescence intensities across the excitation spectrum. However, fluorescence intensities were up to 6.7‐ and 9‐fold higher for *Li* pHin2^*Lm*^ and *Lm* pHin2^*Lm*^ compared to *Lm* pHin depending on excitation wavelength and pH (Figure 2). This is in line with data showing about 8‐fold higher fluorescence for pHluorin2 over pHluorin when expressed in eukaryotic cells (Mahon, 2011).

**Figure 2:**
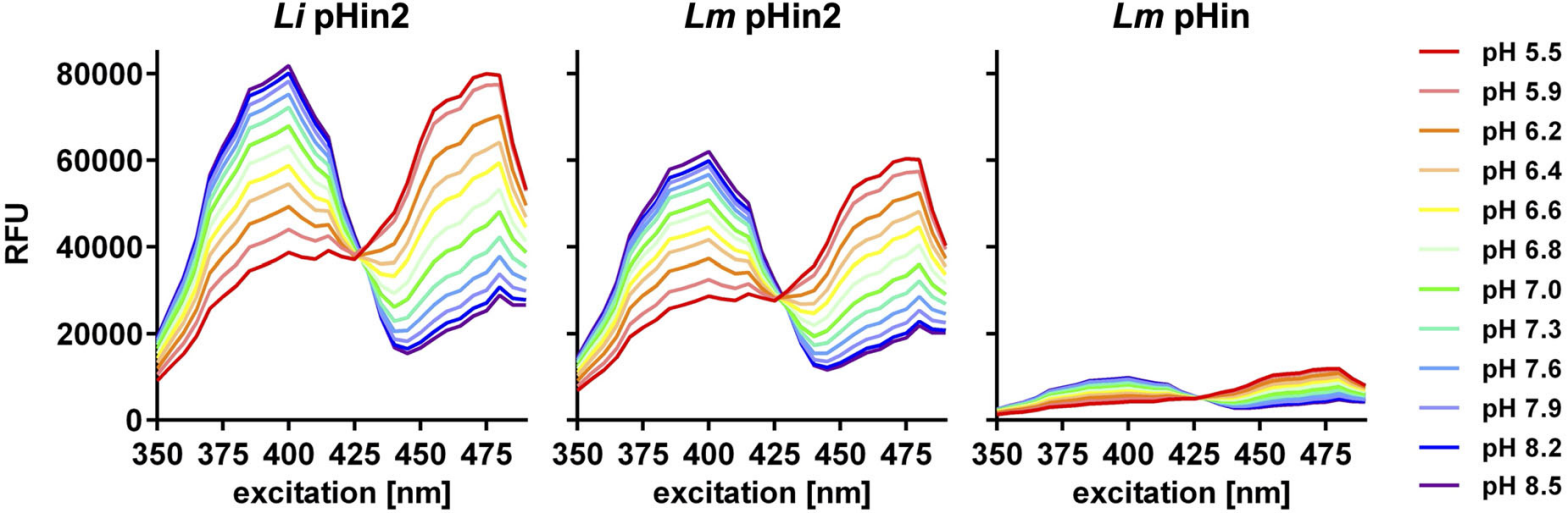
Relative fluorescence units at 520 nm (RFU) across a spectrum of excitation wavelengths (350–490 nm) of *Listeria innocua* LMG2785/pNZ‐pHin2^*Lm*^ (*Li* pHin2; left), *Listeria monocytogenes* EGDe/pNZ‐pHin2^*Lm*^ (*Lm* pHin2, middle), or EGDe/pNZ P_help_‐pHluorin (*Lm* pHin, right). Bacteria were resuspended in LMB adjusted to the indicated pH and permeabilized with cetyltrimethylammonium bromide (0.002%). Values are means of *n* = 3 independent cultures per strain.

To further demonstrate that the new biosensors behave similarly to the previously published strain, dose–response experiments were performed with nisin A and pediocin PA‐1 (both purchased from Sigma‐Aldrich), two antimicrobial peptides that kill target bacteria by disrupting membrane integrity (Brötz et al., 1998; Chikindas et al., 1993). In particular, 2‐fold dilutions of the peptides were prepared in microtiter plates, starting with concentrations of 10 µg/ml nisin and 1.25 µg/ml pediocin. The sensor strains were prepared as described above and 100 µl aliquots were added to bacteriocin dilutions. After incubation for 30 min at room temperature in the dark, fluorescence at 510 nm was measured after excitation at the two maxima (400 and 470 nm) and the ratio 400/470 was calculated (Figure 3). This ratio is an indicator of the average cell integrity across the entire population of sensor bacteria in the well of the microtiter plate. All three strains had comparable dose–response curves and showed a complete shift in excitation ratios at concentrations of 2.5 µg/ml of nisin and 625 ng/ml of pediocin, respectively (Figure 3). Furthermore, the pHin2 sensor strains showed a higher dynamic range of excitation ratios between ∼0.9 (for disrupted cells) and around ∼2.5 (intact), compared to ∼0.6 to ∼1.5 for *Lm* pHin. This may be an advantage for measurements of samples with high background fluorescence, for example, supernatants of bacteria grown in complex media.

**Figure 3:**
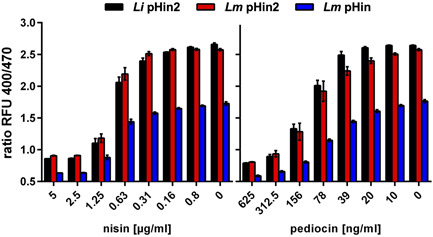
Ratios of fluorescence intensities at 510 nm after excitation at 400 and 470 nm (ratio RFU 400/470) of *Listeria innocua* LMG 2785/pNZ‐pHin2^*Lm*^ (*Li* pHin2; black bars), *Listeria monocytogenes* EGDe/pNZ‐pHin2^*Lm*^ (*Lm* pHin2, red bars) or EGDe/pNZ P_help_‐pHluorin (*Lm* pHin, blue bars) in LMB containing nisin (left) or pediocin (right) at the indicated concentrations. All values are mean ± standard deviation (SD) of *n* = 3 independent cultures per strain.

All three biosensor strains were further analyzed by flow cytometry using an Amnis® CellStream® device (Luminex) equipped with 405 and 488 nm lasers allowing excitation close to the two maxima of pHluorin proteins. For analysis of biosensor bacteria, flow speed was set to “slow,” and laser powers were 10% (forward scatter, FSC; side scatter, SSC), 35% (405 nm), and 40% (488 nm). Bacteria were prepared and treated as described above, subsequently diluted 1:50 in PBS pH = 6.2 and 50 µl of the suspension were then analyzed. The gating strategy (Figure 4a) was as follows: (i) bacterial cells were identified based on FSC and SSC; (ii) bacteria were then gated for singlet events based on the FSC aspect ratio; (iii) singlet events (min. 10,000 per sample) were analyzed for fluorescence intensity (in arbitrary units; AU) at 528 nm for both excitation lasers (405 and 488 nm, respectively). This revealed that >98% of the (singlet) bacteria showed bright fluorescence at 528 nm when excited with either the 405 or the 488 nm laser confirming homogenous expression of pHluorin2 by *Li* pHin2 (Figure 4a). Similar results were obtained for *Lm* pHin2 (Figure A1). All three biosensor strains showed a single population with homogenous fluorescence in the 405/528 nm (excitation/emission) channel (Figure 4b). Flow cytometry confirmed about 8‐fold higher fluorescence of *Li* pHin2 (mean fluorescence intensity; MFI = 38,507 ± 2098AU; *N* = 3 independent cultures) and *Lm* pHin2 (MFI = 37,886 ± 530 AU) in the 405/528 nm channel compared to *Lm* pHin (MFI = 4143 ± 515 AU), which is in line with plate reader measurements (Figure 2). Additionally, flow cytometry was performed on untreated and nisin‐treated (10 µg/ml, 30 min) *Li* pHin2 biosensors (Figure 4c) using PBS pH = 6.2 as sheath fluid. This allowed us the detection of biosensor bacteria in clearly distinct gates according to the 405/528 and 488/528 nm channels depending on the treatment. Bacteria in these two gates either represent untreated, intact, or nisin‐treated, membrane‐disrupted bacteria, respectively. This demonstrates that flow cytometry can be used to assess intracellular pH and in consequence membrane integrity of pHluorin‐expressing sensor bacteria on a single‐cell level.

**Figure 4:**
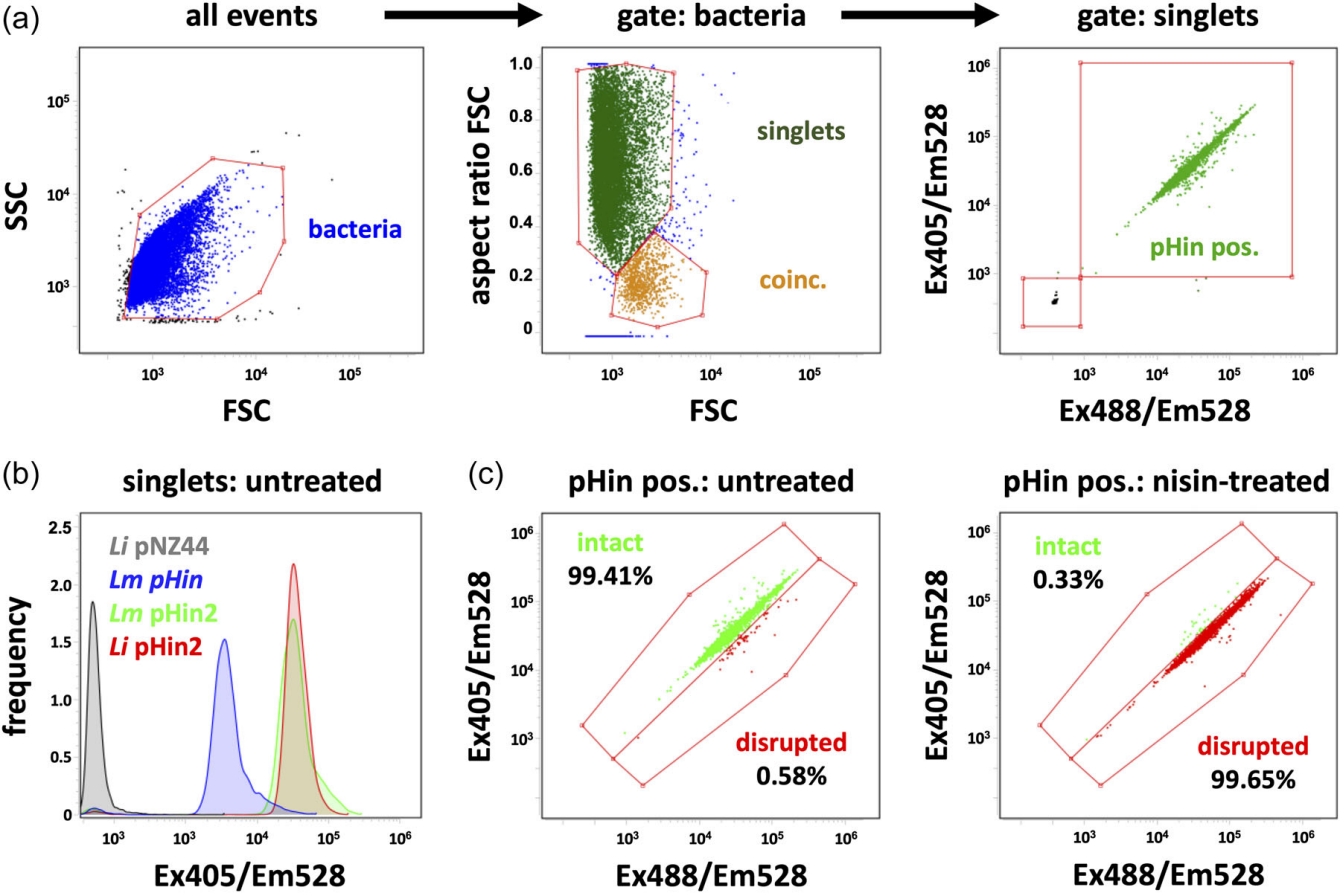
Analysis of *Listeria* spp. biosensor strains by flow cytometry. (a) Gating strategy to identify single bacterial cells that express pHluorin (pHin pos.). Among all recorded events, bacteria were gated based on their forward and side scatter (FSC, SSC; left panel). Singlets were identified by plotting the FSC aspect ratio over the FSC (middle) and pHluorin (pHin pos.) singlets were analyzed for fluorescence intensity (emission wavelength 528 nm) after excitation with the 405 nm (Ex405/Em528) and 488 nm laser right (Ex488/Em528). (b) Histogram plot of fluorescence intensity in the singlet gate of *Lm* pHin (yellow), *Lm* pHin2 (red), *Li* pHin2 (green), and the empty vector control strain *Li* pNZ44 (black). (c) Dot plots of fluorescence intensity from the Ex405/Em528 and Ex488/Em528 channels of untreated *Li* pHin2 (left panel) or after incubation with nisin (10 µg/ml, 30 min; right panel), allowing discrimination between bacteria with intact or disrupted plasma membranes, respectively.

In conclusion, we provide two new biosensors of the genus *Listeria* that allow the analysis of membrane damage using the ratiometric pH‐dependent fluorescent protein pHluorin2 (Mahon, 2011). Both strains show up to 9‐fold higher fluorescence compared to previously published strain *L. monocytogenes* EGDe/pNZ‐P_help_‐pHluorin (Crauwels et al., 2018). All three strains behave comparable regarding challenges with membrane‐damaging chemicals and peptides. The improved fluorescence properties of the new strains may facilitate analysis in matrices with high background fluorescence, where the BSL1 strain *L. innocua*/pNZ‐pHin2^*Lm*^ can be used when BSL2 strains are not allowed. Moreover, they were shown to be suitable for single‐cell analysis of membrane integrity by flow cytometry.

## AUTHOR CONTRIBUTIONS

**Sebastian J. Reich**: Conceptualization (equal); data curation (equal); formal analysis (lead); methodology (equal); supervision (equal); validation (equal); visualization (equal); writing—original draft (equal); writing—review and editing (equal). **Jonas Stohr**: Formal analysis (supporting). **Oliver Goldbeck**: Methodology (supporting); supervision (equal); validation (equal); writing—review and editing (equal). **Bastian Fendrich**: Formal analysis (supporting). **Peter Crauwels**: Methodology (equal); supervision (equal); validation (equal). **Christian U. Riedel**: Conceptualization (equal), data curation (equal), funding acquisition (lead); methodology (equal); project administration (lead); resources (lead); supervision (equal); validation (equal); visualization (equal); writing—original draft (equal); writing—review and editing (equal).

## CONFLICT OF INTEREST

None declared.

## ETHICS STATEMENT

None required.

## Figures and Tables

**Figure A1:**
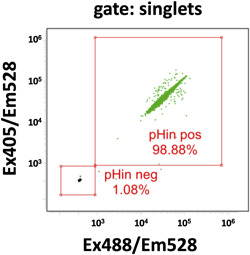
Flow cytometry analysis of fluorescence of singlet population of the *Listeria monocytogenes*/pNz‐pHin2Lm sensor strain

## Data Availability

The data generated or analyzed during this study are included in the published article. The sequence of pNZ‐pHin2^*Lm*^ is available in GenBank, accession number ON668434: https://www.ncbi.nlm.nih.gov/nuccore/ON668434.

## 1

 Figure A1
